# Dynamic inter-glycan interactions regulate site-specific *N*-glycan maturation in human Fcγ receptor III

**DOI:** 10.1093/glycob/cwag075

**Published:** 2026-09-14

**Authors:** Yue Zhang, Hirokazu Yagi, Masako Okina, Yutaka Hashimoto, Koichi Kato, Takumi Yamaguchi

**Affiliations:** School of Materials Science, Japan Advanced Institute of Science and Technology, 1-1 Asahidai, Nomi 9231292, Japan; Graduate School of Pharmaceutical Sciences, Nagoya City University, 3-1 Tanabe-dori, Mizuho-ku, Nagoya 467-8603, Japan; Exploratory Research Center on Life and Living Systems (ExCELLS), National Institutes of Natural Sciences, 5-1 Higashiyama, Myodaiji-cho, Okazaki 444-8787, Japan; Graduate School of Medical Sciences, Nagoya City University, 1 Kawasumi, Mizuho-cho, Mizuho-ku, Nagoya 467-8601, Japan; Graduate School of Medical Sciences, Nagoya City University, 1 Kawasumi, Mizuho-cho, Mizuho-ku, Nagoya 467-8601, Japan; Graduate School of Pharmaceutical Sciences, Nagoya City University, 3-1 Tanabe-dori, Mizuho-ku, Nagoya 467-8603, Japan; Exploratory Research Center on Life and Living Systems (ExCELLS), National Institutes of Natural Sciences, 5-1 Higashiyama, Myodaiji-cho, Okazaki 444-8787, Japan; Institute for Molecular Science, National Institutes of Natural Sciences, 5-1 Higashiyama, Myodaiji-cho, Okazaki 444-8787, Japan; School of Materials Science, Japan Advanced Institute of Science and Technology, 1-1 Asahidai, Nomi 9231292, Japan; Graduate School of Pharmaceutical Sciences, Nagoya City University, 3-1 Tanabe-dori, Mizuho-ku, Nagoya 467-8603, Japan; Exploratory Research Center on Life and Living Systems (ExCELLS), National Institutes of Natural Sciences, 5-1 Higashiyama, Myodaiji-cho, Okazaki 444-8787, Japan

**Keywords:** Fcγ receptor III, inter-glycan interaction, molecular dynamics simulation, *N*-glycan maturation, site-specific glycosylation

## Abstract

Protein *N*-glycosylation generates diverse glycoforms that influence protein structure and function, yet the molecular rules governing site-specific glycan maturation remain incompletely understood. Accumulating evidence indicates that glycosylation outcomes are shaped by determinants embedded within glycoproteins themselves, beyond enzyme availability alone. Here, we investigate how neighboring glycans modulate site-specific *N*-glycan maturation in human Fcγ receptor III (FcγRIII). Building on previous glycoproteomic studies showing restricted processing of the N45 glycan in FcγRIII molecules, we combined reciprocal glycosylation-site mutagenesis with molecular dynamics simulations. Introduction of an N64 glycosylation sequon into FcγRIIIa reduced N45 glycan maturation, whereas removal of the N64 sequon from FcγRIIIb produced the reciprocal effect. Simulations using glycan models, analyzed primarily by contact maps and contact frequencies, showed that the N45 glycan dynamically contacts neighboring N64 and N169 glycans. These findings support a model in which the isoform-specific N64 glycan, in addition to the adjacent N169 glycan shared by both FcγRIII isoforms, contributes to reduced N45 glycan maturation by reshaping the local glycan environment around N45.

## Introduction

Protein *N*-glycosylation is inherently heterogeneous, giving rise to diverse glycoforms that critically influence protein structure and function. Although the presence of consensus sequons and the cellular repertoire of glycosylation enzymes provide the biochemical framework for *N*-glycan attachment and processing, these factors alone do not fully explain the pronounced site-specific heterogeneity observed in many glycoproteins.

Large-scale site-specific glycoproteomic analyses have established that the structural context of individual glycosylation sites—including solvent accessibility and local tertiary environment—is a major determinant of *N*-glycan type, core fucosylation, and branching (Thaysen-Andersen and Packer 2012). In addition, peptide motifs and sequence-dependent trafficking can bias glycosylation outcomes by regulating enzyme recognition or access to Golgi-resident glycosylation enzymes (Saito et al. 2022; Yagi et al. 2024). Thus, glycosylation outcomes are encoded by multiple layers of information within glycoproteins.

In parallel, recent studies have highlighted that spatially constrained or dense glycosylation can restrict enzymatic access through steric and dynamic effects, leading to the selective retention of under-processed glycans, most prominently in highly glycosylated viral envelope proteins (Pritchard et al. 2015; Watanabe et al. 2020). Molecular dynamics (MD) simulations further suggest that protein structure modulates glycan exposure by selecting accessible conformational ensembles rather than imposing static constraints (Fogarty and Fadda 2021). Whether such inter-glycan interactions contribute to site-specific glycan maturation in physiological mammalian receptors has remained unclear.

**Figure 1:**
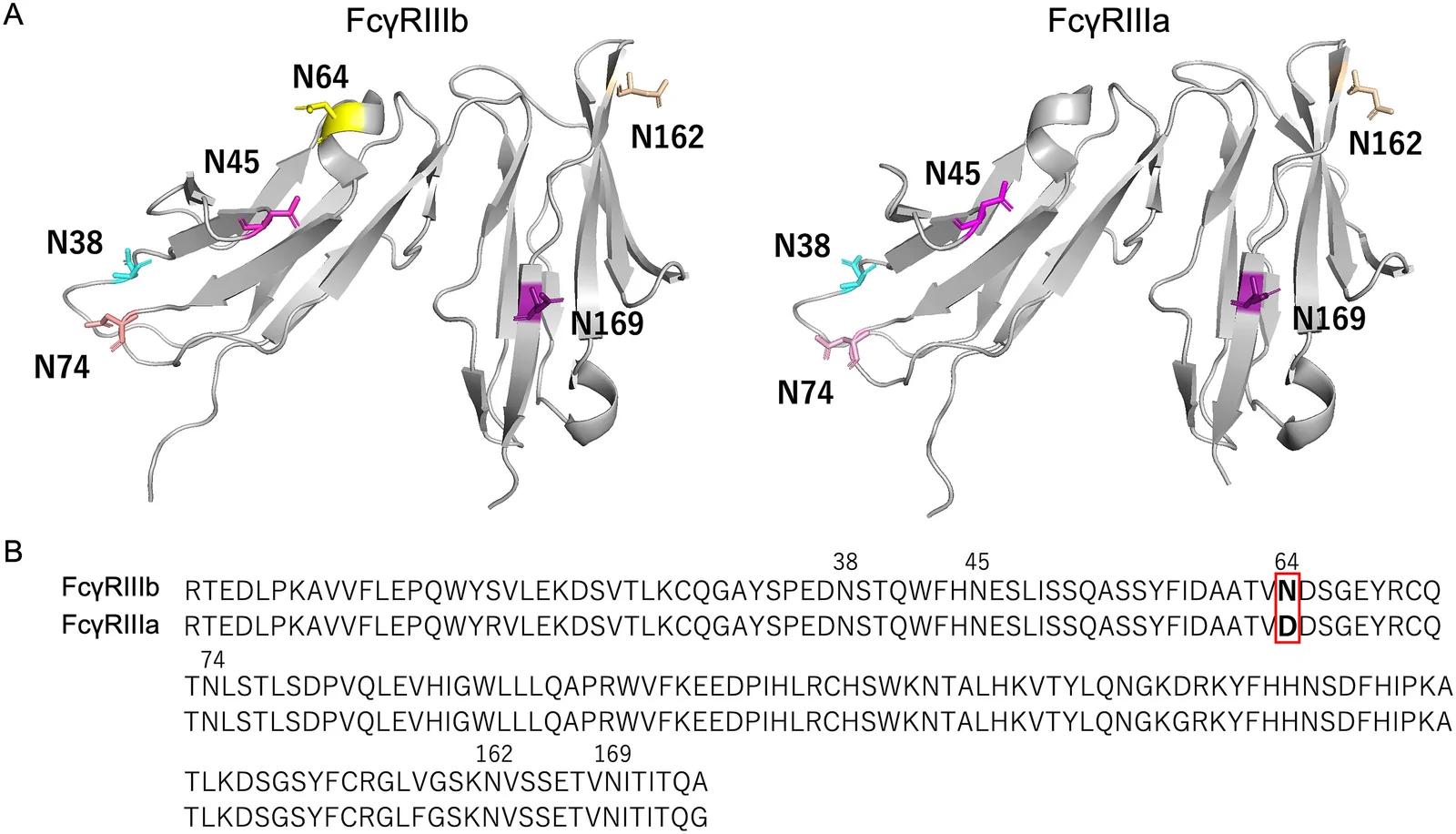
Comparison of *N*-glycosylation sites in human FcγRIII isoforms. (A) Crystal structures of the extracellular domains of human FcγRIIIb (PDB: 1FNL) and FcγRIIIa (PDB: 5ML9). (B) Sequence alignment of the extracellular domains of FcγRIIIb and FcγRIIIa. *N*-glycosylation sites are indicated by stick models and highlighted.

Human Fcγ receptors provide an informative system to address this question. FcγRIIIa and FcγRIIIb, also known as CD16a and CD16b, are closely related low-affinity IgG Fc receptors with highly similar extracellular domains but distinct cellular distributions and membrane anchoring modes (Bruhns 2012). FcγRIIIa is expressed mainly on natural killer cells and macrophage/monocyte lineages as a transmembrane receptor, whereas FcγRIIIb is expressed on neutrophils as a GPI-anchored receptor. Previous site-resolved analyses have consistently shown that, in FcγRIIIb, one particular *N*-glycosylation site exhibits a relative immaturity compared with other sites within the same receptor, whereas FcγRIIIa does not display a pronounced site-specific suppression of glycan maturation at the corresponding position (Kawasaki et al. 2014; Yagi et al. 2018; Pavan et al. 2024). Notably, FcγRIIIb uniquely contains an additional *N*-glycosylation site absent from FcγRIIIa, and structural models place this site in close spatial proximity to the relatively immature glycan (Fig. 1). These observations raise the possibility that inter-glycan interactions contribute to the isoform-dependent difference in glycan maturation.

Here, we integrate site-specific glycan analyses with MD simulations to define how this local glycan environment regulates N45 maturation. We show that N64-dependent changes in N45 glycan maturation correlate with dynamic contacts among neighboring glycans, revealing inter-glycan interactions as an additional structural layer in the glycosylation program.

## Results and discussion

To elucidate the molecular basis of the isoform-dependent difference in N45 glycan maturation, we examined how the additional N64 glycan in FcγRIIIb influences the local glycan environment. Prior site-specific analyses showed that FcγRIIIb contains a relatively under-processed N45 glycan compared with the corresponding site in FcγRIIIa (Kawasaki et al. 2014; Yagi et al. 2018; Pavan et al. 2024), prompting us to test whether local glycan architecture contributes to this selective immaturity.

Human FcγRIIIb contains multiple *N*-glycosylation sites, including N38, N45, N64, N74, N162, and N169, each occupying a distinct structural and functional context (Ferrara et al. 2011; Kawasaki et al. 2014). The N162 glycan at the Fc-binding interface enhances receptor-antibody interactions through carbohydrate-carbohydrate contacts with Fc glycans, whereas the N45 glycan is positioned outside this interface and can negatively influence Fc binding, mainly through steric effects (Ferrara et al. 2006; Shibata-Koyama et al. 2009; Mizushima et al. 2011; Sakae et al. 2017).

**Figure 2:**
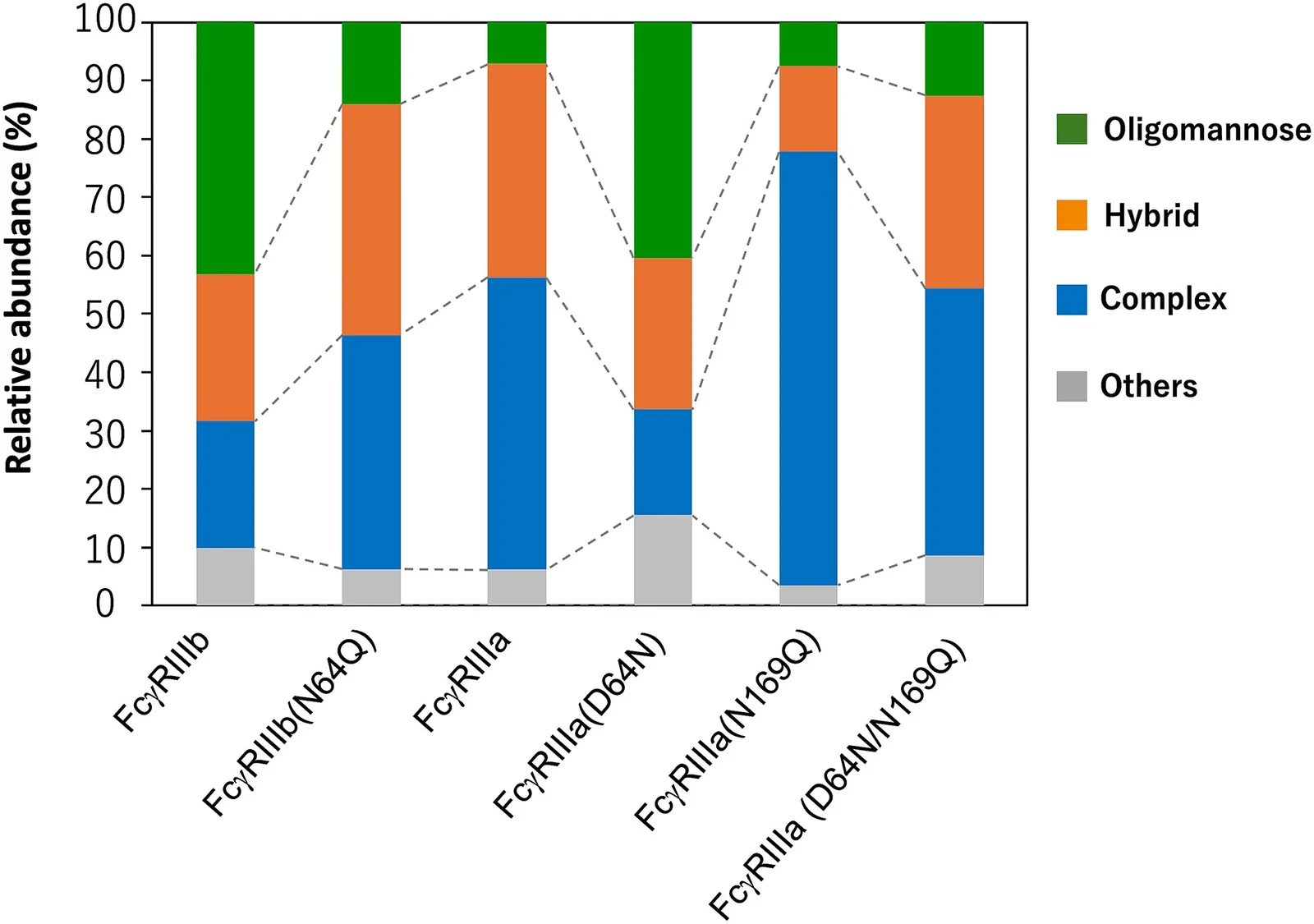
Site-specific *N*-glycan profiling of FcγRIII variants by LC–MS/MS. Relative abundances of N45 glycoform classes are shown for wild-type FcγRIIIa, FcγRIIIa(D64N), FcγRIIIa(N169Q), FcγRIIIa(D64N/N169Q), wild-type FcγRIIIb, and FcγRIIIb(N64Q). N45 glycoforms were grouped into oligomannose-, hybrid-, and complex-type classes to compare the effect of neighboring glycosylation sites. Glycopeptide identification results generated using Proteome Discoverer (PD) with the Byonic search engine are provided in Supplementary Table S1. Relative abundances were calculated from normalized precursor ion intensities of the major N45-containing glycopeptide. The glycoprofiles are based on a single LC–MS/MS measurement for each sample and are presented for descriptive comparison only.

The N64 glycosylation site is present in FcγRIIIb but absent from FcγRIIIa (Fig. 1), providing an opportunity to test whether a spatially proximal glycan can further modulate N45 maturation. We first introduced an *N*-glycosylation sequon at position 64 in FcγRIIIa (D64N), thereby mimicking this local feature of FcγRIIIb (Fig. 2 and Supplementary Table S1). In the N45 profiles, evaluated as descriptive glycoprofiles based on single LC–MS/MS measurements, wild-type FcγRIIIa was dominated by complex- and hybrid-type classes (86.7%) with a smaller oligomannose-type fraction (7.2%), whereas FcγRIIIa(D64N) shifted toward oligomannose-type and hybrid-type species (40.5% and 25.9%, respectively). Conversely, wild-type FcγRIIIb showed a high oligomannose-type fraction at N45 (43.2%), and removal of the N64 sequon from FcγRIIIb by the N64Q mutation shifted the N45 profile toward complex- and hybrid-type species (79.8%) with a reduced oligomannose-type fraction (14.0%).

Because N169 represents another neighboring glycosylation site within the local glycan environment around N45, we also analyzed FcγRIIIa(N169Q) and FcγRIIIa(D64N/N169Q) to place the N64 effect in this broader context. FcγRIIIa(N169Q) retained a highly processed N45 profile (89.1% complex- and hybrid-type species), whereas FcγRIIIa(D64N/N169Q) showed an intermediate profile (78.9% complex- and hybrid-type, 12.6% oligomannose-type, and 8.6% truncated/intermediate species). These reciprocal and combinatorial mutational results support the conclusion that the N64 glycan is an important isoform-specific local modulator of N45 maturation, rather than the sole determinant of N45 under-processing.

**Figure 3:**
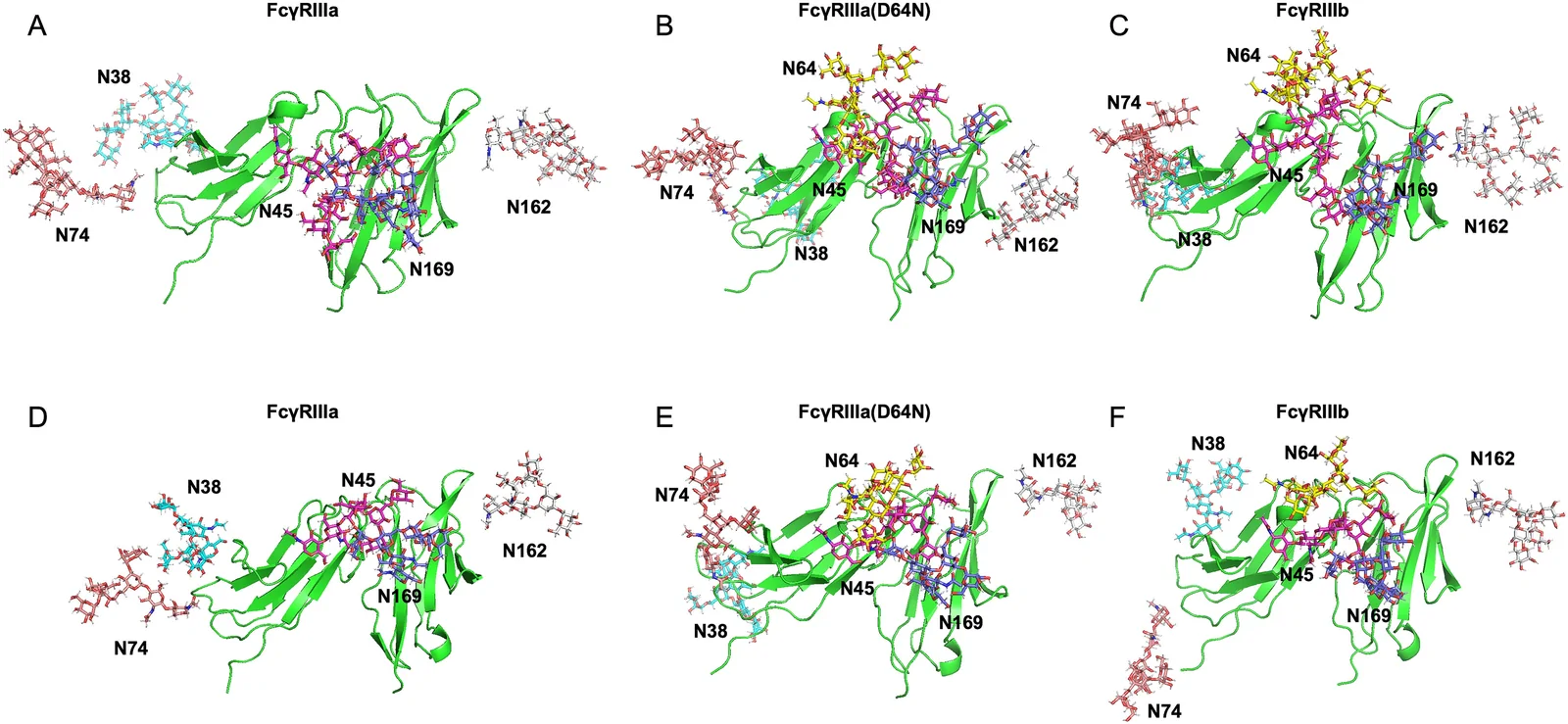
Structural overview of the local glycan environment around N45. Representative models show the spatial relationship of the N45 glycan with neighboring glycans, including N169 in all models and N64 in FcγRIIIa(D64N) and wild-type FcγRIIIb. Panels A–C show M8B models, and panels D–F show Man5 models, for wild-type FcγRIIIa, FcγRIIIa(D64N), and wild-type FcγRIIIb. The *N*-glycans at N38, N45, N64, N74, N162, and N169 are labeled and visually distinguished consistently across panels to highlight the N45-centered local glycan environment.

**Figure 4:**
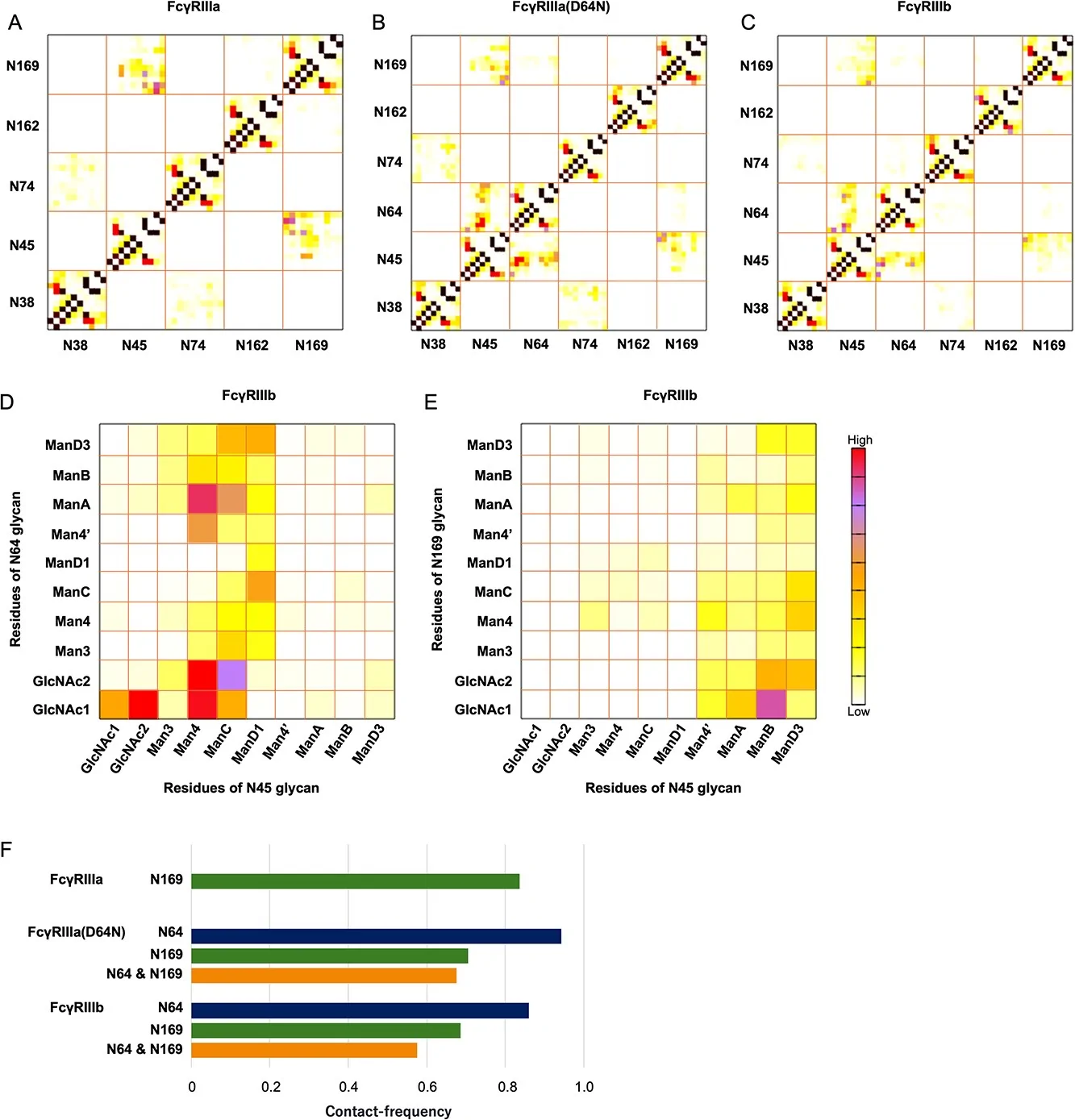
Contact-map analysis of N45-neighboring glycan interactions in the M8B models. (A–C) Overall contact maps across glycosylation sites in FcγRIIIa, FcγRIIIa(D64N), and FcγRIIIb. Source data are provided in Supplementary Table S2. (D and E) Detailed views showing interactions between N45 and N64 glycans (D) and between N45 and N169 glycans (E) in FcγRIIIb. (F) Quantification of contact frequencies between N45 and neighboring glycans.

We next used MD simulations to examine whether neighboring glycans dynamically contact the N45 glycan during conformational sampling. Because the purpose of these simulations was to evaluate local accessibility during glycan processing rather than to reconstruct the final experimental glycoform distribution, we analyzed both M8B models and Man5 models that represent a more trimmed oligomannose-processing intermediate (Fig. 3 and Supplementary Fig. S1). We then performed contact map and contact-frequency analyses. In the M8B simulations, N45-N64 contacts were observed at high frequency in FcγRIIIa(D64N) and FcγRIIIb (0.94 and 0.86 by glycan, respectively), whereas wild-type FcγRIIIa, which lacks the N64 glycan, served as the corresponding negative control (Fig. 4, Supplementary Fig. S2 and Supplementary Table S2). The N45 glycan also contacted the N169 glycan, including in wild-type FcγRIIIa, indicating that N45 resides in a pre-existing local glycan environment that is further reshaped by the presence of the N64 glycan. Similar N45-neighboring glycan contacts were observed in the Man5 simulations, in which N45-N64 contact frequencies were 0.97 in FcγRIIIa(D64N) and 0.87 in FcγRIIIb, supporting the relevance of this interaction pattern to a more processed intermediate state. Hydrogen-bond analysis supported the contact-map results (Supplementary Table S3), while SASA analyses further indicated that the glycan also makes contact with the receptor surface (Supplementary Fig. S3).

Together, these results identify N64 as an isoform-specific local modulator of N45 maturation in recombinant or engineered FcγRIII systems. Introduction of the N64 sequon reduced N45 glycan maturation, N64 removal shifted N45 in the opposite direction, and MD simulations showed high-frequency N45-N64 contacts. However, N45 maturation is not governed by N64 alone: N45 also contacts N169, and the FcγRIIIa(N169Q) and FcγRIIIa(D64N/N169Q) profiles indicate that N169 contributes to the broader local constraint rather than acting as a stand-alone switch.

This broader interpretation is consistent with recent analyses of FcγRIII glycosylation from distinct biological sources. FcγRIIIb isolated from neutrophils lacks detectable occupancy at the N38 and N64 sequons yet retains a predominantly oligomannose-rich N45 glycan, whereas soluble or recombinant FcγRIIIb preparations can display distinct site-specific glycan profiles (Wojcik et al. 2020). Similarly, FcγRIIIa, which lacks the N64 sequon, contains substantial oligomannose and hybrid-type glycans at N45 in primary-cell-derived material (Roberts et al. 2020). MD simulations by Fogarty and Fadda further suggest that the FcγRIIIa protein surface can restrict the accessible conformations of the N45 Man5 glycan, especially the α(1-6) arm (Fogarty and Fadda 2021). Thus, N45 maturation appears to be limited by an intrinsic local structural environment, while neighboring glycans such as N169 can further shape this environment.

These findings extend the established paradigm in which protein structural context governs site-specific glycosylation (Thaysen-Andersen and Packer 2012; Mathew et al. 2021) by showing that neighboring glycans can also shape the local accessibility of a processing-sensitive site. Spatially constrained glycan arrangements are known to restrict Golgi processing in densely glycosylated viral envelope proteins, as exemplified by the HIV-1 Env mannose patch and oligomannose clusters in coronavirus spike glycoproteins (Pritchard et al. 2015; Watanabe et al. 2020), and our results suggest that a related local principle can operate in a mammalian immune receptor. Together with previous demonstrations that amino-acid sequences can encode glycosylation determinants through direct enzyme recognition (Saito et al. 2022) or regulation of intracellular trafficking pathways (Yagi et al. 2024), our results identify dynamic inter-glycan contacts as an additional protein-intrinsic layer within the glycosylation program.

Incorporating glycan dynamics and inter-glycan regulation into models of glycosylation will therefore be essential for achieving a mechanistically grounded understanding of site-specific glycoform predictability.

## Materials and methods

### Expression and purification of soluble FcγRIII variants

Synthetic genes encoding soluble human FcγRIIIa variants (wild-type, D64N, N169Q, and D64N/N169Q) and FcγRIIIb variants (wild-type and N64Q), precloned into the mammalian expression vector pcDNA3.4, were purchased from Thermo Fisher Scientific. The constructs were transiently transfected into Expi293 cells using the ExpiFectamine 293 Transfection Kit (Thermo Fisher Scientific) according to the manufacturer’s instructions. Cells were cultured in Expi293 Expression Medium at 37 °C in a humidified incubator with 8% CO_2_ and shaking at 125 rpm. Culture supernatants were harvested 4 days post-transfection by centrifugation and filtration.

Recombinant FcγRIII proteins containing a C-terminal hexahistidine tag were purified from the supernatant using a Ni2+-affinity column (Roche) under native conditions. Clarified supernatants were loaded onto the column equilibrated with binding buffer (20 mM sodium phosphate, 150 mM NaCl, pH 7.4), washed extensively, and eluted with an imidazole-containing buffer (20 mM sodium phosphate, 150 mM NaCl, 300 mM imidazole, pH 7.4). Eluted fractions were dialyzed against 20 mM sodium phosphate and 150 mM NaCl (pH 7.4) and then concentrated using Amicon Ultra centrifugal ultrafiltration devices with a 10 kDa molecular weight cutoff.

### Proteolytic digestion and glycopeptide enrichment

Purified FcγRIII proteins were reduced with 10 mM dithiothreitol at 56 °C for 30 min and alkylated with 20 mM iodoacetamide at room temperature in the dark for 30 min. The samples were then digested sequentially with chymotrypsin (Promega, Madison, WI, USA) at an enzyme-to-substrate ratio of 1:50 and incubated overnight at 37 °C to generate glycopeptides suitable for site-specific glycosylation analysis. The resulting peptide mixtures were desalted using a MonoSpin C18 spin column (GL Sciences, Tokyo, Japan) and subjected to LC–MS/MS analysis.

### LC–MS/MS analysis

The peptides were analyzed using a Vanquish Neo UHPLC system equipped with a C18 column (Nikkyo Technos, Tokyo, Japan, NTCC-360/75-3-125), a FAIMS Pro Duo interface, and an Orbitrap Exploris 240 mass spectrometer (Thermo Fisher Scientific, Waltham, MA, USA). Peptides were separated with solvent A (0.1% formic acid in water) and solvent B (80% acetonitrile containing 0.1% formic acid), using gradients from 6% to 31% B over 30 min and 31% to 50% B over 5 min at 300 nL/min. FAIMS compensation voltages were set at −45 and −65 V. Full MS/data-dependent MS^2^ spectra were acquired over m/z 400–2,000 at resolutions of 120,000 and 30,000, respectively.

### Data analysis

Glycopeptide identification and glycan assignment were performed using Proteome Discoverer (Thermo Fisher Scientific) in conjunction with Byonic (version 4.3; Protein Metrics Inc., Cupertino, CA, USA). Searches were conducted against a glycan database containing 132 human *N*-glycans. Glycopeptide searches were performed assuming chymotryptic digestion with a maximum of two missed cleavages, a precursor mass tolerance of 10 ppm, and a fragment ion mass tolerance of 0.5 Da. Carbamidomethylation of cysteine residues was specified as a fixed modification.

### Quantification and classification of N45 glycoforms

Relative N45 glycoform abundances were calculated using precursor ion intensities obtained from Proteome Discoverer in conjunction with Byonic. For quantification, only the major N45-containing glycopeptide was used, whereas alternative peptide forms, including missed-cleavage peptides and other minor peptide variants, were excluded from the analysis. The precursor ion intensity of each glycoform was normalized to the summed intensity of all quantified N45 glycoforms detected within the corresponding sample and expressed as a percentage of the total N45 glycan signal. Glycoforms without quantitative intensity values were regarded as not detected and were excluded from the normalization.

Glycans were classified according to their monosaccharide compositions. Glycans with compositions of HexNAc(2)Hex(5–10) were classified as oligomannose-type glycans, whereas glycans with compositions of HexNAc(3)Hex(4–10)Fuc(0–2)NeuAc(0–4) were classified as hybrid-type glycans. Glycans containing HexNAc(4) or more were classified as complex-type glycans regardless of fucosylation or sialylation. Glycan structures that did not meet these criteria were grouped as “Others,” representing truncated or intermediate glycan structures that could not be assigned to the three major glycan classes.

The glycoprofiles shown in Fig. 2 were generated from a single LC–MS/MS measurement for each sample. Therefore, biological replicates and statistical analyses were not performed, and the results are presented as descriptive glycoprofiles.

### Molecular dynamics simulations

All simulations were performed using the GENESIS software package (Jung et al. 2024) with the CHARMM36m force field and the TIP3P water model. Data analyses were conducted using the CPPTRAJ program in the Amber package (Roe and Cheatham 3rd. 2013). All computations were performed at the JAIST Research Center for Advanced Computing Infrastructure.

The atomic coordinates of FcγRIIIb were taken from a crystal structure of the extracellular domains of human FcγRIIIb (PDB ID: 1FNL). The structures of FcγRIIIa and its D64N variant were modeled based on the 1FNL structure using PyMOL. Using CHARMM-GUI, either high-mannose-type M8B (G48369JO) or Man5 (G00028MO) glycans were attached to the *N*-glycosylation sites to build the glycoprotein structures (Jo et al. 2008; Jo et al. 2011). The M8B models were used to compare the positional effect of the N64 glycan under matched glycan-composition conditions, whereas the Man5 models were used to evaluate whether N45-neighboring glycan contacts persist in a more trimmed processing-intermediate state. Each system was solvated in 150 mM NaCl and surrounded by a 10 Å water padding in a rectangular box.

All bonds involving hydrogen atoms were constrained using the SHAKE algorithm, and water molecules were kept rigid using the SETTLE algorithm. Long-range electrostatic interactions were evaluated using the PME method. Nonbonded interactions were truncated at a cutoff distance of 12 Å, with a switching function applied from 10.0 Å. The system was first energy-minimized and equilibrated for 1 ns in the *NVT* ensemble (300 K), followed by equilibration for 500 ps in the *NPT* ensemble (300 K, 1 atm) using a 2 fs time step. To allow a longer time step, hydrogen mass repartitioning was applied to the solute, adjusting the solute hydrogen mass to approximately 3 amu. The time step was increased to 3.5 fs, and the system was further equilibrated for 1.05 ns in the *NPT* ensemble.

To enhance sampling, the generalized replica-exchange with solute tempering (gREST) method was employed (Kamiya and Sugita 2018). In the gREST method, a selected solute region is virtually heated via Hamiltonian scaling to facilitate conformational transitions, while the solvent remains at the reference temperature. Here, the entire glycoprotein was defined as the solute, and replica exchange was applied to its dihedral, improper, CMAP, partial charge, and Lennard-Jones terms. A total of 64 replicas were used with solute effective temperatures spanning 300–500 K. Each replica was first equilibrated for 1.05 ns at its assigned temperature, followed by production simulations totaling 73.5 ns in the *NVT* ensemble.

All data analyses were performed using the last 63 ns (3,000 frames) of the 300 K trajectories. A residue-residue contact was defined if the minimum distance between atoms of two residues was less than 3 Å. Glycan-level contact frequencies were calculated from the fraction of frames in which at least one residue pair between two glycans satisfied this criterion. Hydrogen bonds were analyzed using standard distance and angle criteria, and the SASA for each glycan was calculated with the LCPO method. SASA analyses were used as supplementary descriptors of accessibility, whereas contact maps and contact frequencies were used as the principal readouts of local glycan proximity.

## Supplementary Material

Supplementary_Information_cwag075

Supplementary_TableS1_cwag075

Supplementary_TableS2_cwag075

## Data Availability

The mass spectrometry data for protein identification have been deposited to jPOSTrepo (Okuda et al. 2025) under the ID JPST004516.
