# Supplementary material for: Dynamic inter-glycan interactions regulate site-specific N-glycan maturation in human Fcγ receptor III
Source: Glycobiology. 2026 Sep 14;36(10):cwag075. doi: 10.1093/glycob/cwag075 (PMC13580079; doi:10.1093/glycob/cwag075)
Supplement: Supplementary file 1 [file supplementary_information_cwag075.pdf]

Supplementary Information

Dynamic inter-glycan interactions regulate site-specific *N*-glycan maturation in human Fcγ receptor III

Yue Zhang[1,†], Hirokazu Yagi[2,3,†], Masako Okina[4], Yutaka Hashimoto[4], Koichi Kato[2,3,5]*, Takumi Yamaguchi[1,2,3]*

[1] School of Materials Science, Japan Advanced Institute of Science and Technology, 1-1 Asahidai, Nomi 9231292, Japan

[2] Graduate School of Pharmaceutical Sciences, Nagoya City University, 3-1 Tanabe-dori, Mizuho-ku, Nagoya 467-8603, Japan

[3] Exploratory Research Center on Life and Living Systems (ExCELLS), National Institutes of Natural Sciences, 5-1 Higashiyama, Myodaiji-cho, Okazaki 444-8787, Japan

[4] Graduate School of Medical Sciences, Nagoya City University, 1 Kawasumi, Mizuho-cho, Mizuho-ku, Nagoya 467-8601, Japan

[5] Institute for Molecular Science, National Institutes of Natural Sciences, 5-1 Higashiyama, Myodaiji-cho, Okazaki 444-8787, Japan

[†] These authors contributed equally to this work.

*Corresponding authors: Koichi Kato, kkatonmr@ims.ac.jp; Takumi Yamaguchi, takumi@jaist.ac.jp

Supplementary Table S1. Protein Discoverer–Byonic glycopeptide identification and quantification results for FcγRIII variants.

The worksheets contain the complete Protein Discoverer–Byonic output for each FcγRIII variant analyzed. Rows highlighted in green indicate the major Asn45-containing glycopeptides used for quantification in Figure 2. Relative N45 glycoform abundances were calculated using the Protein Discoverer Abundance values by normalizing the abundance of each quantified N45 glycoform to the summed abundance of all quantified N45 glycoforms within the corresponding sample. Only the major Asn45-containing glycopeptide was used for quantification, whereas alternative peptide forms, including missed-cleavage peptides, were excluded.

Supplementary Table S2. Numerical source data for contact maps of glycans.

Supplementary Table S3. Hydrogen-bond analysis of N45-neighboring glycan interactions.

	M8B			Man5	
	N45-N64	N45-N169		N45-N64	N45-N169
FcγRIIIa	-	55%		-	19%
FcγRIIIa(D64N)	68%	45%		41%	31%
FcγRIIIb	30%	24%		23%	22%

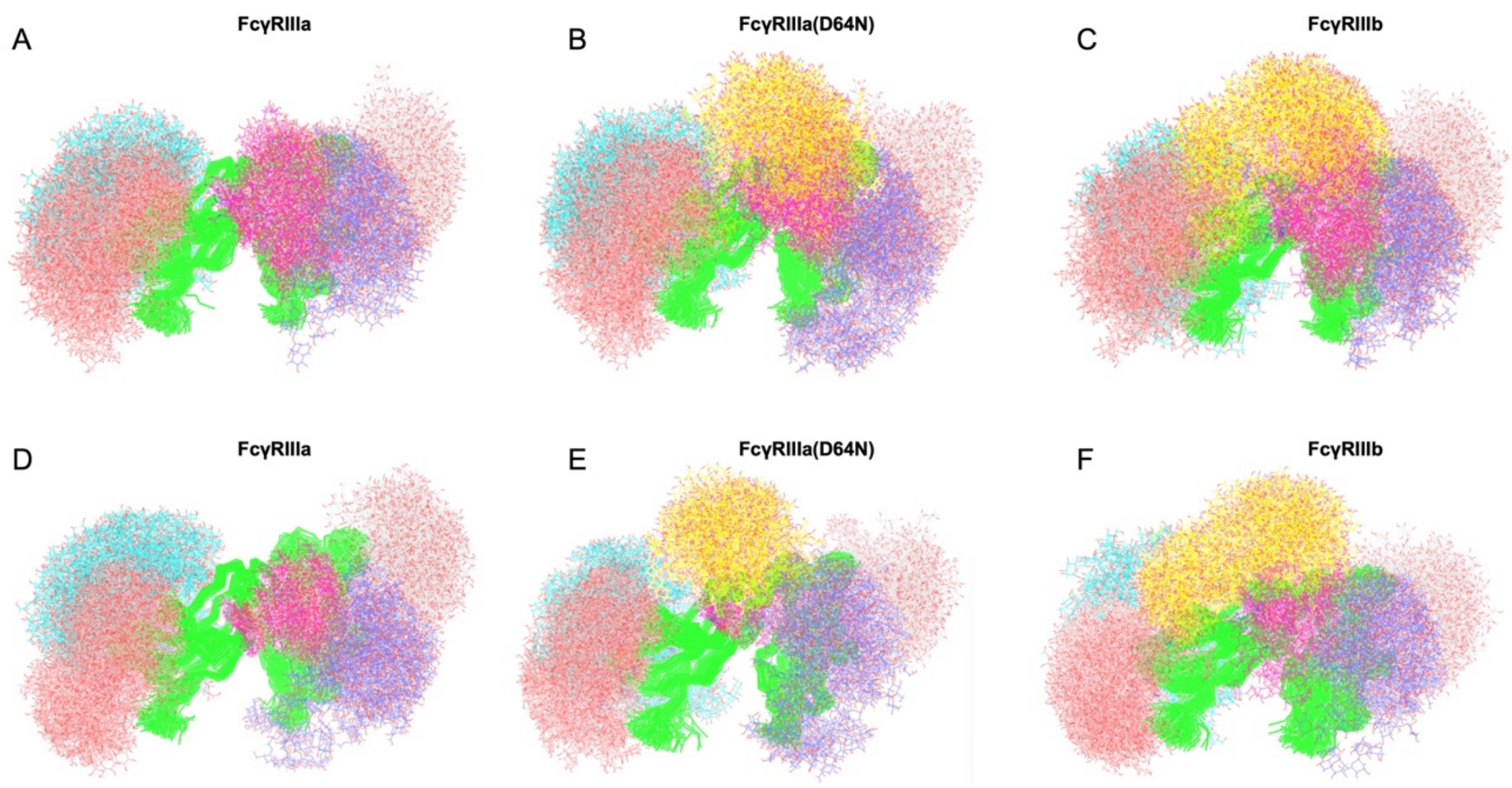

Supplementary Figure S1. Conformational ensembles of N45-neighboring glycans in FcγRIII models. Ensemble views from MD simulations show the spatial sampling of the N45 glycan and neighboring glycans around the FcγRIII surface. Panels A-C show M8B models, and panels D-F show Man5 models, for wild-type FcγRIIIa, FcγRIIIa(D64N), and wild-type FcγRIIIb.

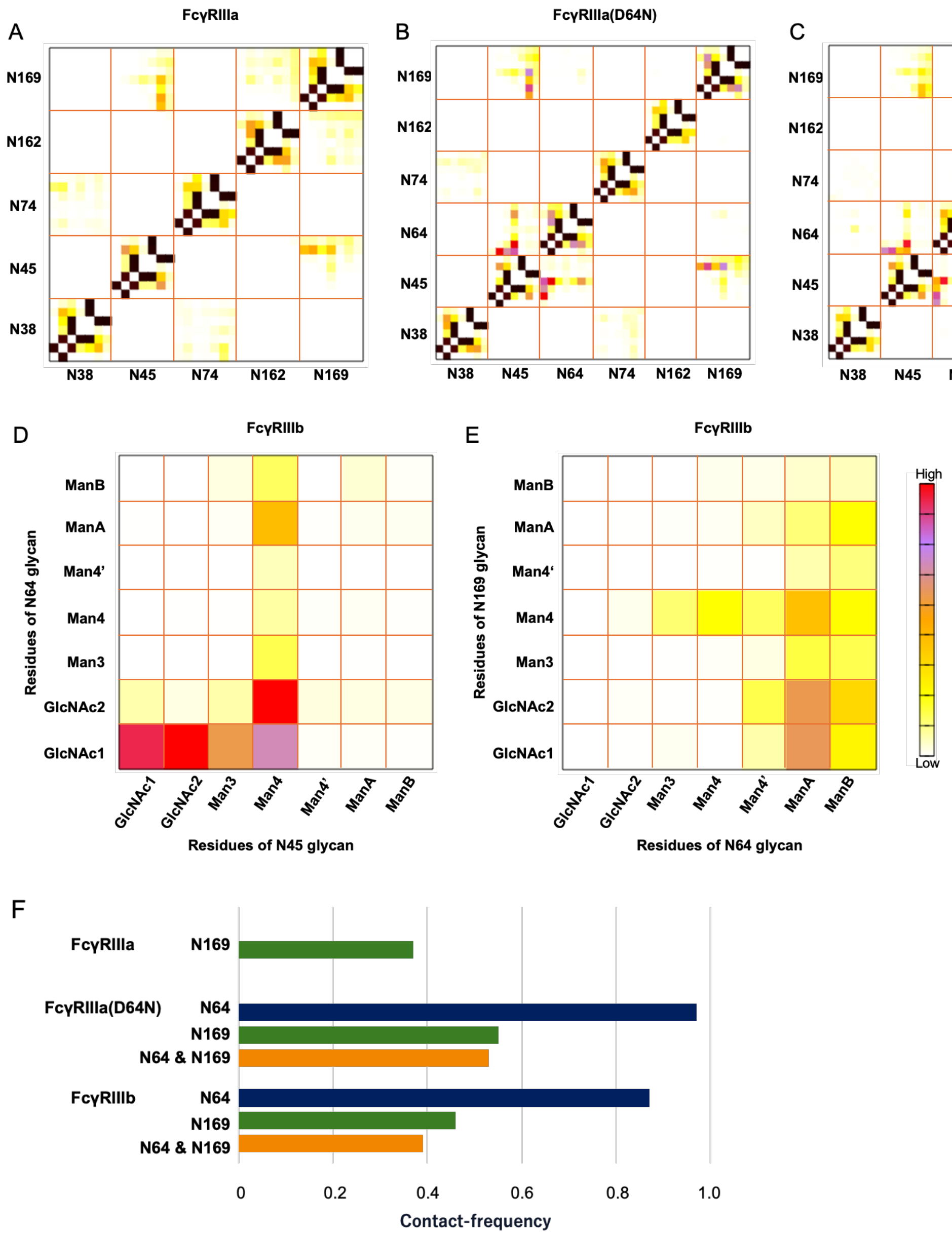

Supplementary Figure S2. Contact-map analysis of N45-neighboring glycan interactions in the Man5 models. (A–C) Overall contact maps across glycosylation sites in FcγRIIIa, FcγRIIIa(D64N), and FcγRIIIb. Source data are provided in Supplementary Table S2. (D, E) Detailed views showing interactions between N45 and N64 glycans (D) and between N45 and N169 glycans (E) in FcγRIIIb. (F) Quantification of contact frequencies between N45 and neighboring glycans.

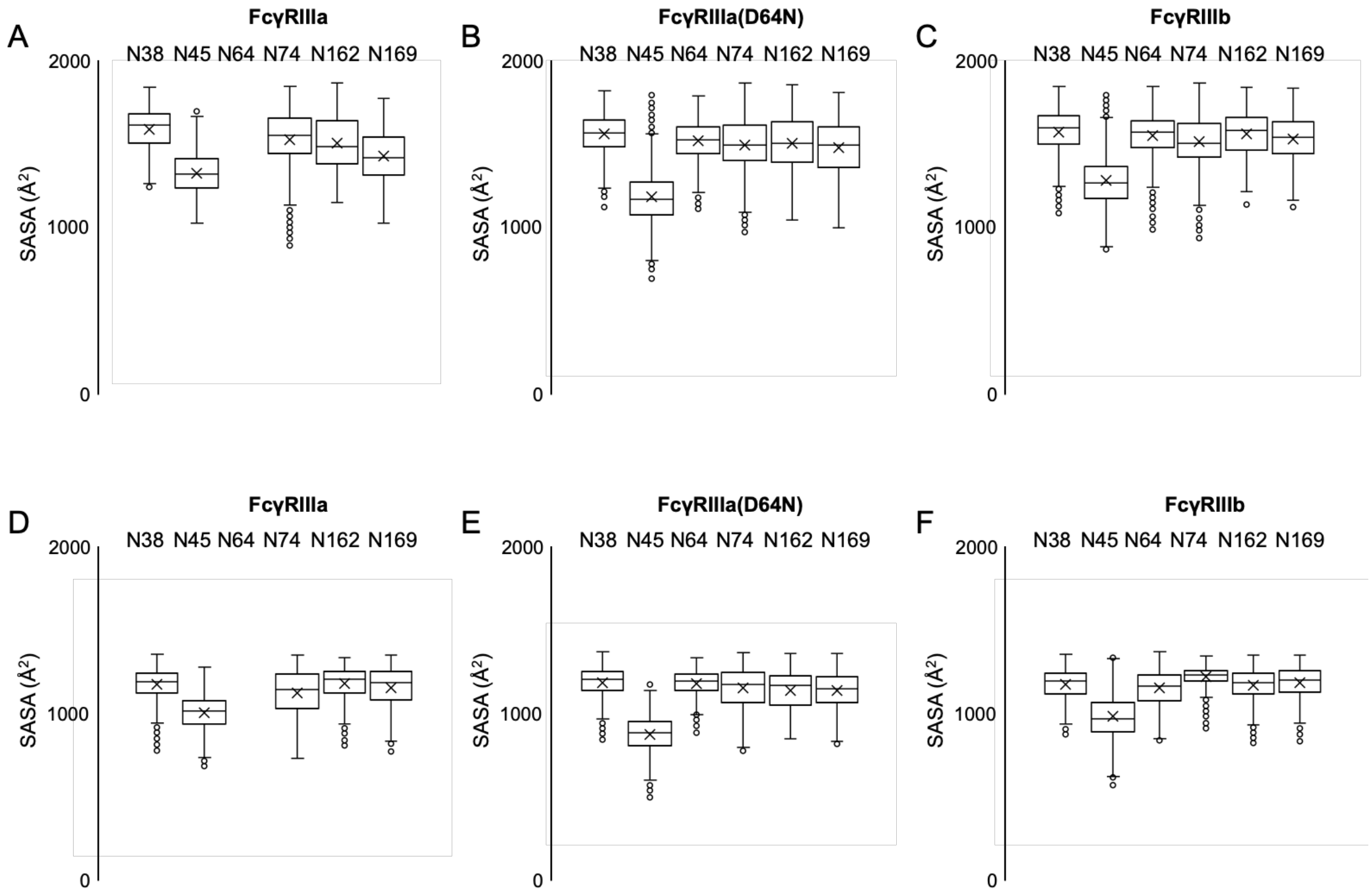

Supplementary Figure S3. Solvent-accessible surface area analysis of FcγRIII glycans. Solvent-accessible surface area (SASA) values were calculated for glycans in wild-type FcγRIIIa, FcγRIIIa(D64N), and wild-type FcγRIIIb (A–C) in M8B and (D–F) Man5 models.
